# Measurement of leaf lamina moisture with a low-cost electrical humidity sensor: case study on a wheat *water*-mutant

**DOI:** 10.1186/s12870-019-1987-4

**Published:** 2019-10-07

**Authors:** Agata Rascio, Michele Rinaldi, Giuditta De Santis, Nicola Pecchioni, Gabriele Palazzo, Nicola Palazzo

**Affiliations:** 1 Council for Agricultural Research and Economics, Research Centre for Cereal and Industrial Crops, S.S. 673 Km 25,200, 71122 Foggia, Italy; 2 Palazzo Sistemi Elettronici (PSE) S.r.l., Via Mione, 39, 71122 Foggia, Italy

**Keywords:** Leaf moisture, Electrical sensor, Wheat breeding, Bound water

## Abstract

**Background:**

The presence and persistence of water on the leaf can affect crop performance and thus might be a relevant trait to select for or against in breeding programmes. Low-cost, rapid and relatively simple methods are of significant importance for screening of large populations of plants for moisture analysis of detached leaves. Leaf moisture can be detected using an electric circuit, where the resistance changes are proportional to the moisture of the measured surface. In this study, we present a protocol to analyse genotypic differences through the electrical properties of living or stored tissues, performed using a commercial device. Expanded and non-expanded leaves were compared to determine the effects of leaf maturity on these data. Two wheat genotypes that differ in tissue affinity for bound water were used to define the influence of water status.

**Results:**

The device indirectly estimates leaf moisture content using two electrodes applied to the leaf lamina of fresh and stored samples. Single moisture readings using this moisture meter had mean execution time of ~ 1.0 min. Exponential associations provided good fits for relationships between the moisture meter reading (MMR) and the electrical resistance applied to the electrodes. MMR normalised for the water/ dry matter ratio (MMRnorm) was lower for mature leaves of the *water*-mutant than those of wild-type, for the fully hydrated fresh leaves. MMR of fully mature leaves when partially dehydrated and measured after 10 min at 27 °C and 40% relative humidity was greater for the *water*-mutant than the wild-type.

**Conclusions:**

This case study provides a low-cost tool to compare electrical-resistance estimates of leaf moisture content, together with a promising and rapid phenotyping protocol for genotypic screening of wheat under standard environmental conditions. Measurement of changes in MMR with time, of fresh and partially dehydrated leaves, or of MMR normalised to tissue water content allowed for differentiation between the genotypes. Furthermore, the differences observed between genotypes that here relate particular to tissue affinity for bound water suggest that not only the free-water fraction, but also other water fractions, can affect these electrically estimated leaf moisture measures.

## Background

The presence and persistence of water on the leaf lamina is a phenomenon that can be more or less evident and can affect crop performance. High persistence of water increases microbe and insect infections [1, 2], ice formation [3], and tissue damage by focusing sun light [4]. Moreover, by increasing the presence of a water film on the leaf surface, poor water repellent leaves can significantly reduce gas exchange and subtract water from root systems [5].

Leaf moisture can be detected measuring the resistance of an applied electric circuit. In some cases, leaf wetness originates from the plant, with different degrees of susceptibility for wetting among plants [6]. Electrical estimates of leaf moisture are affected by environmental conditions, repellency and hydrophilicity of the plant surfaces, anatomical, biochemical, biophysical and functional traits [7–9]. Tissue resistance (e.g., across the cell cytoplasm, vacuoles, cell wall, extracellular spaces) and tissue capacitance (e.g., plasma membrane, tonoplast) ratios can also change with leaf age, and hence with chemical composition of the leaves [10]. In addition, studies from the beginning of the last century on the electrical conductivity of cellulose materials have highlighted that bound water (which has biophysical properties different from free water) can modify the relationships between moisture content of cellulose material and specific electrical conductance [11, 12]. As for wood and also in wheat, there are different types of bound water, as strongly, weakly and very weakly bound, due to different charged groups on the macromolecular surfaces of the cell walls [13].

Considering the potential involvement of leaf moisture in several processes that are related to plant performance and that variability in leaf wettability and surface water retention has been observed for many species [14], phenotyping methods to compare genotypes in terms of their electrical properties might be useful in breeding programmes. To successfully apply selection or to identify molecular markers, the essential pre-requisites are genetic variability and availability of rapid and low-cost reproducible screening methods. Several automatic systems for the analysis of electrical properties are available [15], although they are particularly affected by sensor design, calculation mode and protocol used [16]. Clip sensors are also available, which can measure single leaves in a dew chamber, or through droplet application to intact or detached leaves [17], but these methods are laborious and difficult to apply to many plants.

With the aim of developing a rapid and affordable phenotyping protocol, a commercial, low-cost, electrical resistance sensor designed for wood moisture measurement was tested. Expanded (mature) and not fully expanded (immature) wheat leaves were collected from a durum wheat (*Triticum turgidum* L. var. *durum*) genotype, ‘Trinakria’ (wild-type) and its *water*-mutant that had been selected for high leaf affinity for strongly bound water through a simplified analysis of the water thermodynamic properties [18]. The *water*-mutant has also been shown to differ from its wild-type in terms of nuclear magnetic resonance imaging of proton relaxation times and the histochemical characteristics of its cell walls [19]. Furthermore, the electrical properties of full turgid, partially dehydrated and dried re-humidified leaves were examined. Indeed, high-throughput analysis of breeding lines, recombinant inbred lines or near isogenic lines at fixed plant growth phases requires the processing of many samples (immediately after collection) over a short time. So, by using dried samples, the possibility to postpone the genotypic comparisons to some days after sample collection was also examined.

## Results

### Relationship between the MMR and the applied electrical resistance

**Fig. 1:**
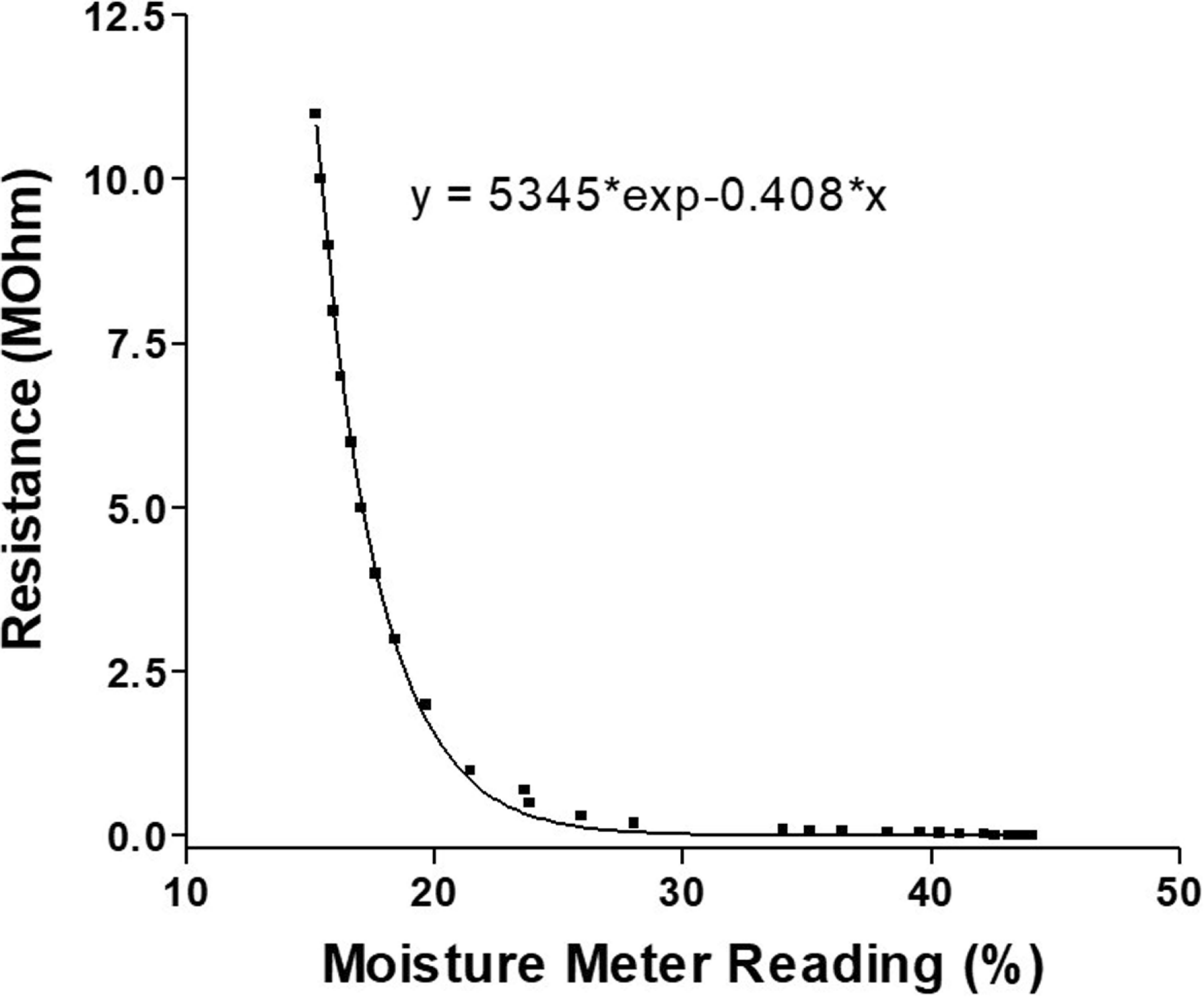
The empirical dependence of the MMR on the electrical resistances. A flux millivoltmeter and a resistivity box were mounted in parallel to the electrodes. Increasing resistances were applied and the corresponding direct current voltage signal and MMRs were recorded. The equation of the interpolating curve is shown

Figure 1 shows the moisture values as displayed by the moisture meter as a function of the electrical resistance applied to the electrodes. Satisfactory repeatability of measurements was observed, with a maximum deviation of ±2% at the environmental temperature of 20 °C. An exponential association fit well to the experimental data (degrees of freedom, 25; R^2^, 0.9987). The data points between the MMRs of 20 and 30 were above the line, which suggested that resistance values calculated by interpolation in this range were slightly underestimated (by the order of 0.1 Mohm).

**Fig. 2:**
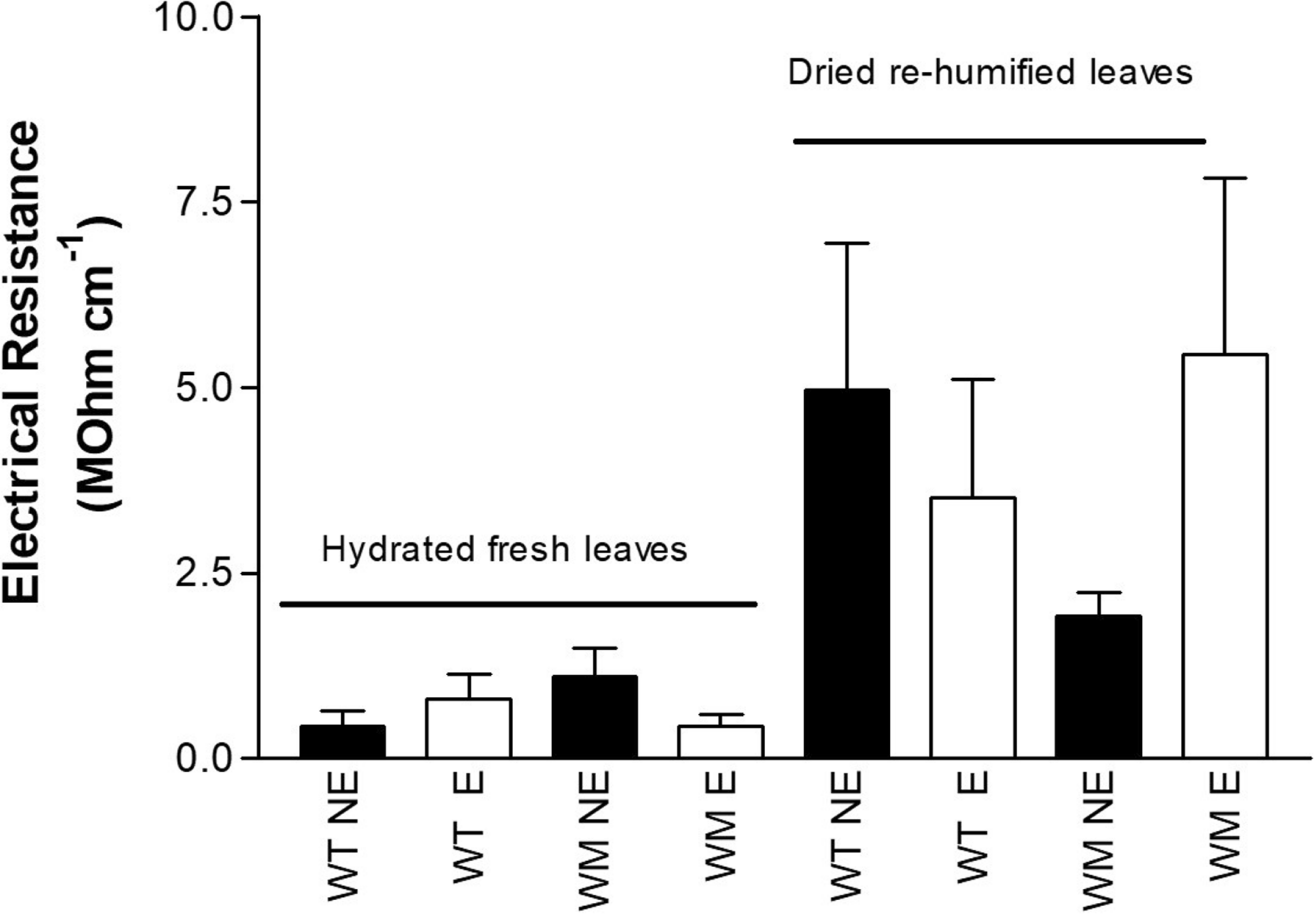
Estimated electrical resistance of the hydrated fresh and dried re-humidified leaves, according to genotype and leaf expansion. Data are means ±standard error. WT, wild-type; WM, water-mutant; NE, non-expanded leaves; E, expanded leaves

Figure 2 shows the mean interpolated electrical resistance of the hydrated fresh and dried re-humidified leaves according to genotype (i.e., wild-type, *water*-mutant) and leaf maturity (i.e., expanded, non-expanded), as estimated using the exponential curve shown in Fig. 1. The electrical resistance of the hydrated fresh leaves showed the same magnitude as reported for fresh maize leaves [20], and was about one tenth of the dried re-humidified leaves. Within each sample type (i.e., hydrated fresh, dried re-humidified leaves) there were no significant differences between genotypes or leaf ages (Table 1).

**Table 1:** ANOVA results for the differences among the genotypes (i.e., wild-type, *water*-mutant), leaf maturity (i.e., expanded, non-expanded) and their interactions for leaf electrical resistance, gravimetric moisture content (dry weight basis), moisture meter readings (MMRs) and MMR normalised (MMRnorm) to tissue water content

Parameter	Units of	Leaf sample	Genotypes		Leaf expansion		Interaction	
	measure		F	P	F	P	F	P
Electrical resistance	MOhm cm^−1^	Fresh	0.3	0.617	0.3	0.609	3.2	0.080
		Re-humidified	0.1	0.758	0.3	0.566	1.9	0.174
Gravimetric moisture content	%	Fresh	1.1	0.303	0.1	0.757	1.6	0.216
		Re-humidified	0.8	0.366	2.7	0.1143	1.2	0.284
MMR	a.u. (nominal %)	Fresh	0.7	0.409	0.0	0.974	2.7	0.111
		Re-humidified	0.0	0.852	0.0	0.990	1.2	0.280
MMRnorm	% a.u. (gH_2_O gDW^−1^)^−1^	Fresh	3.9	0.056	0.6	0.460	5.1	0.031*
		Re-humidified	0.5	0.471	7.0	0.012*	4.9	0.034*

*, significant (*P* < 0.05)

### MMRs and whole leaf water content of hydrated fresh and dried re-humidified leaves

**Fig. 3:**
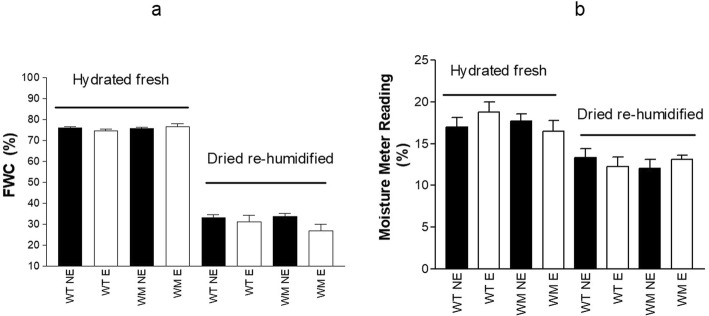
Percentage whole-leaf moisture contents (dry weight basis), measured according to the gravimetric method (**a**) as compared to the readings of the Powerfix moisture meter (**b**), for hydrated fresh and dried re-humidified leaves according to genotype and leaf expansion. Data are means ±standard error. WT, wild-type; WM, water-mutant; NE, non-expanded leaves; E, expanded leaves

Overall, the gravimetric moisture contents of the whole leaves ranged from about 300% for hydrated fresh leaves to 50% for dried re-humidified leaves (Fig. 3). On the basis of the moisture meter readings, the sample moisture ranged from 18% a.u. for the hydrated fresh leaves to 12% a.u. for dried re-humidified leaves. Within each sample type (i.e., hydrated fresh or dried re-humidified), there were no significant differences between genotypes or leaf ages (Table 1), for both gravimetric moisture content and MMRs.

**Fig. 4:**
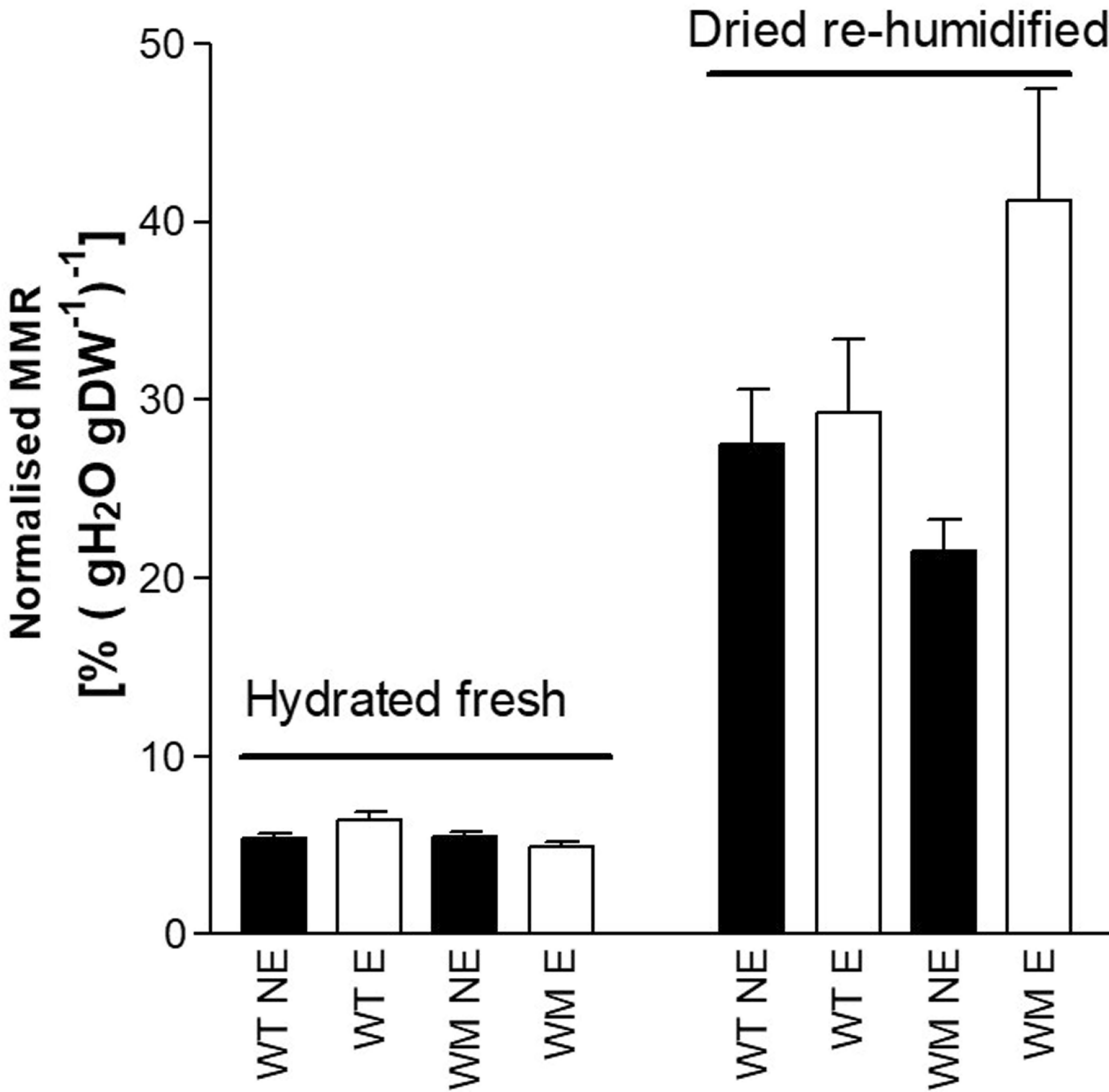
Moisture meter readings normalised (MMRnorm) to the tissue water content (DW basis) of the fresh fully hydrated leaves and the dried re-humidified leaves. WT, wild-type; WM, water-mutant; NE, non-expanded leaves; E, expanded leaves. Data are means ±standard error. Means with the different letters are significantly different (P < 0.05; Tukey’s tests)

The results of ANOVA showed significant *Genotype* × *Leaf maturity* interactions for MMRnorm for both fresh fully hydrated leaves, and for dried re-humidified samples. Indeed, for the fully hydrated fresh leaves, MMRnorm (Fig. 4) was lower for the expanded, mature leaves of the *water*-mutant than the wild-type, while for non-expanded, immature leaves there were no genotypic differences seen. For the re-humidified leaves, MMRnorm for the mature leaves of the *water*-mutant were higher than for its immature leaves, while that of wild-type was not; moreover, ANOVA showed significant *Leaf maturity* effects, because MMRnorm was higher for the expanded leaves.

### Interchangeability of MMR processing on fresh and re-humidified leaves

**Fig. 5:**
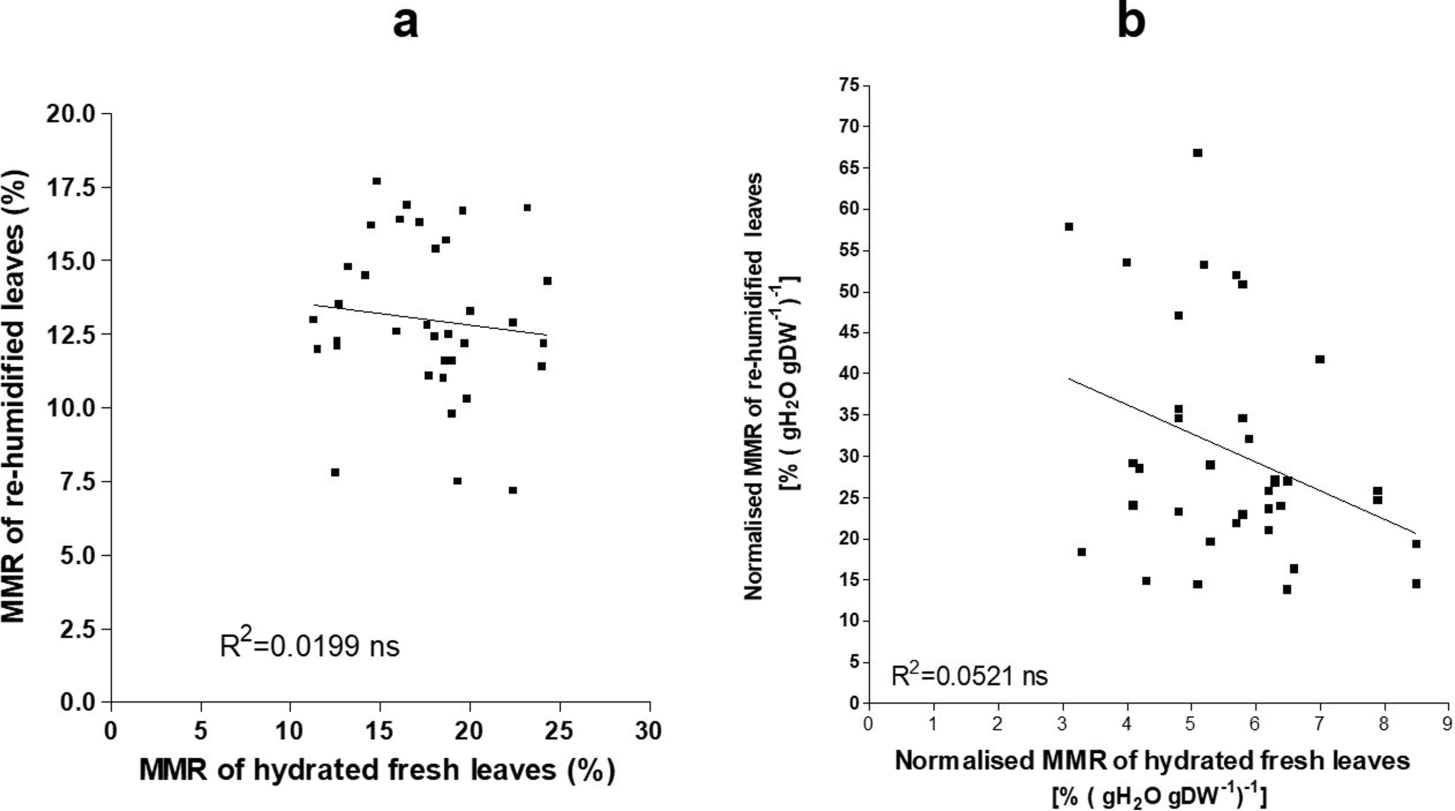
Relationships between the Powerfix moisture meter readings of the leaf of fully hydrated, fresh and dried re-humidified leaves, without normalisation (**a**) and following normalisation (**b**) to the water content (DW basis)

As shown in Fig. 5a, there was no correlation between MMR of the hydrated fresh and dried re-humidified leaves. This lack of correlation indicated that different data can be obtained for MMR, if this is measured for the same hydrated fresh or re-humidified leaves. On the other hand, with MMR normalised (MMRnorm) for tissue moisture content (Fig. 5b), there was an inverse correlation between the hydrated fresh and dried re-humidified leaves (r = − 0.33; P = 0.047).

### MMR changes with time

**Fig. 6:**
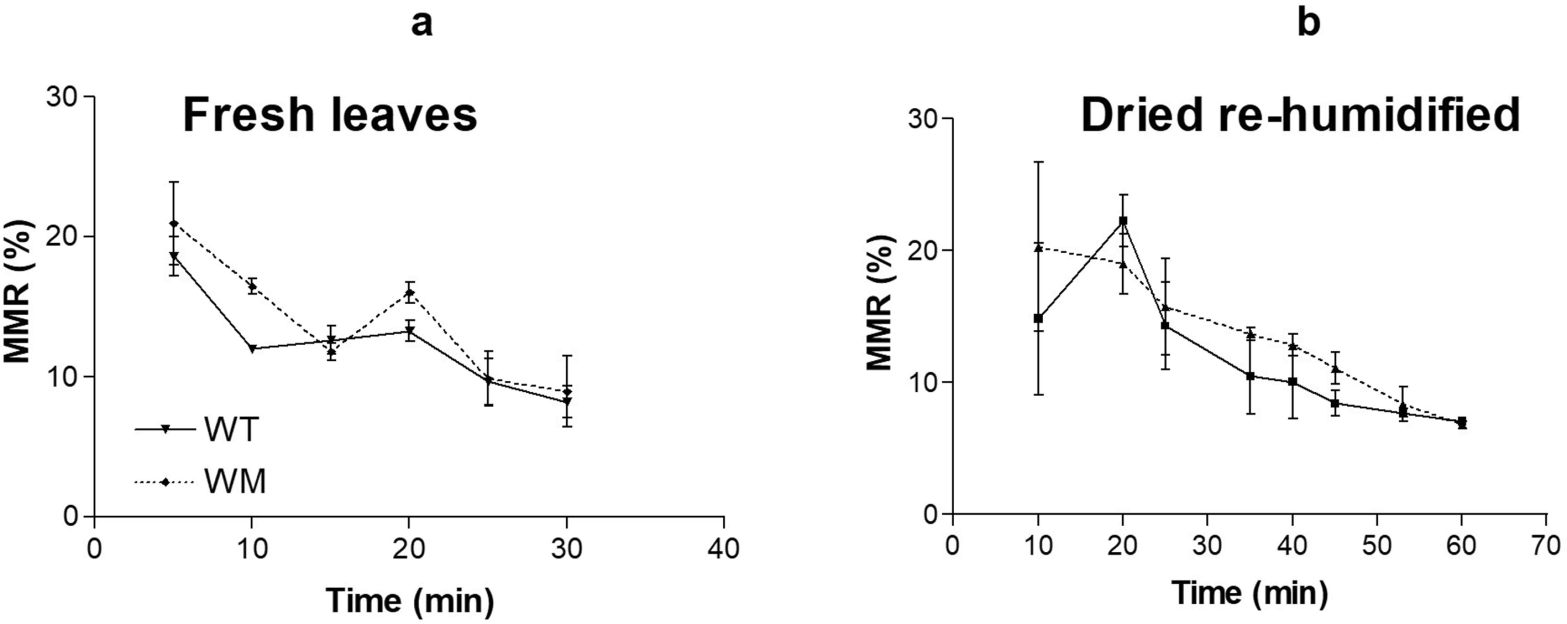
Time-courses of moisture meter readings, for the expanded hydrated, fresh leaves (**a**) and dried re-humidified (**b**) leaves of wild-type (WT; solid line, filled symbols) and water-mutant (WM; dashed line, open symbols). Data are means ±standard error

To define the time interval where MMR changes with time are slower or under steady-state conditions, and thus where the measurements would be less biased by experimental error (Fig. 6) the trend of moisture meter readings with time at 27 °C and 40% relative humidity was examined. The fully expanded hydrated fresh leaves showed an initial rapid MMR decrease over the first 10 min, followed by a slower decrease. Instead, the dried re-humidified leaves initially maintained the MMR, and then showed a rapid reduction from 25 min to 40 min from the beginning of the dehydration process. Across the genotypes, the *water*-mutant generally retained moisture for longer, although this was less clear for the dried re-humidified leaves, as these showed greater standard error of the means. In particular, leaves of the *water*-mutant had significantly higher MMR than those of wild-type at 10 min from the beginning of the dehydration process (Student’s t = 3.748; P = 0.0322; df = 2), when they were partially dehydrated.

## Discussion

Plant traits that reduce water retention of the leaf lamina might affect disease incidence and also water acquisition, use and redistribution [8, 9]. The selective advantages of these traits will vary among species and habitats [21, 22]. Low-cost devices, together with rapid and relatively simple methods, are important for screening of large populations of plants, as required for agronomic evaluation or as part of plant breeding programmes.

The present comparison of hydrated fresh, partially dehydrated and dried re-humidified leaves was designed to evaluate the use of a commercial device with fresh or stored wheat leaves. At the same time, expanded (mature) and non-expanded (immature) leaves were compared to determine any effects of leaf age on these data. Finally, the use of these two genotypes that differ in their tissue affinities for water (i.e., wild-type, *water*-mutant) defined the influence of the water status.

The empirical construction of the MMR versus electrical resistance curves showed that these relative moisture values (as displayed by the moisture meter; a.u.) followed an exponential relationship to the applied electrical resistance. Using this relationship, the leaf resistances estimated appeared to be accurate, as these were of the same magnitude as observed for other cereals [20].

As drying treatments will deeply affected cell organelles, membrane integrity, and bound/free water ratio, and hence tissue resistance and capacitance, the MMR of fully hydrated leaves was significantly greater for the hydrated fresh leaves compared to the dried re-humidified leaves, without any differences within the sample types, in terms of genotype and maturity stage of the leaves. Results were different when the MMR was normalised to the whole leaf water content (i.e., MMRnorm). In this case, MMRnorm was lower for the mature leaves of the *water*-mutant than those of the wild-type, as the fully hydrated fresh leaves. Hence, to eliminate any additive source of experimental error during genotypic screening using the MMR, there is the need for sample standardisation for age and sample type (fresh or re-humidified). Leaf age and genotype affect the tissue electrical properties [10, 22, 23] and chemical composition, and in turn the ratio between the hydrophilic and hydrophobic compounds, the number and type of adsorbing sites on the charged groups of macromolecules, and the tissue affinity for water, and thus the water/ dry matter ratio [13]. Hence, it appears that the electrical properties of the leaves are not closely linked to the structural features of the leaves per se, but rather, they are the consequence of genotype-related and age-related structural changes in the water/dry matter ratio and/or the water properties. ‘Trinakria’ and its *water*-mutant are very close genetically, and their water contents were very similar, with a range from ~ 1.0–3.0 gH_2_O gDW^− 1^ for fully re-humidified and hydrated tissues. Vice versa, the genotypes greatly differ in their tissue affinity for very strongly bound water [18], which amounts in wheat to ~ 0.10 gH_2_O gDW^− 1^ [13] and is hence one tenth to one third that for free water. This can be interpreted as confirmation of the hypothesis [11, 12] that the electrical properties of cellulose materials are related to the bound water and/or to the water/ dry matter ratio, more than to the structural features of the leaves per se. The influence of bound water on the electrical properties of these leaves might also explain why partially dehydrated leaves of the *water*-mutant had lower moisture changes with time (estimated by MMR 10 min from the beginning of the dehydration process), than those of the wild-type. Indeed, over time, the leaf water content decreases together with the free water/ bound water ratio and the number of the free water paths for ionic conductors [24].

## Conclusions

This case study provides a protocol for the use of a commercial moisture meter for rapid and low-cost phenotyping of wheat genotypes under standard environmental conditions. Comparisons of these indoor measurements with outdoor measurements are necessary to validate exportability of these results from the laboratory to the extremely complex and different environmental conditions of the open field. Furthermore, there is the need to compare the relative influences of the genotypes and the environmental variables (including different combinations of temperature, relative humidity, wind intensity, solar radiation) on wetting of wheat leaves, together with the interaction of *Genotype × Environment*.

Our electrical estimates of leaf conductance were carried out indoors on leaves of fixed age, with minimal interference due to changing environmental conditions or leaf maturity stage and showed differences between genetically close genotypes. As a consequence, greater variability can be expected among very distant genotypes. Such a phenotyping protocol using this commercial moisture meter can be applied to define the genetic variability, genetic basis and heritability of electrical estimates of leaf moisture contents before the implementation of relevant plant breeding programmes. The increased affinity for water was the main feature for which the *water*-mutant was selected here, and hence these data indicate that despite the low levels of bound water in an organism, together with the free water, the water affinity might be responsible for the differences in the electrical estimates of wetness.

## Methods

### Description of samples

Seeds of the durum wheat variety ‘Trinakria’ (i.e., wild-type control) and its ‘water-mutant’ were derived from the CREA-CI (Council for Agricultural Research and Economics - Research Centre for Cereal and Industrial Crops) collection. In winter 2016, for each genotype, a 10 m2 plot was sown with 400 seeds/m2, in the experimental fields of CREA-CI (Foggia, Southern Italy). The agronomic practices used were those standard for the crop and area [25]. The plants were fertilised at stem elongation and were grown without irrigation. At the heading stage, 10 expanded leaves below the flag leaf and 10 non-expanded flag leaves (i.e., at about 70% full expansion) were randomly collected from each plot. The leaves were brought to the laboratory and fully hydrated for 2 h in distilled water. Alternatively, for the ‘dried re-humidified’ samples, the leaves were initially dried in an oven at 60 °C for 12 h, and then left for 3 days in a dryer at 20 °C and 90% relative humidity.

### Moisture meter readings

**Fig. 7:**
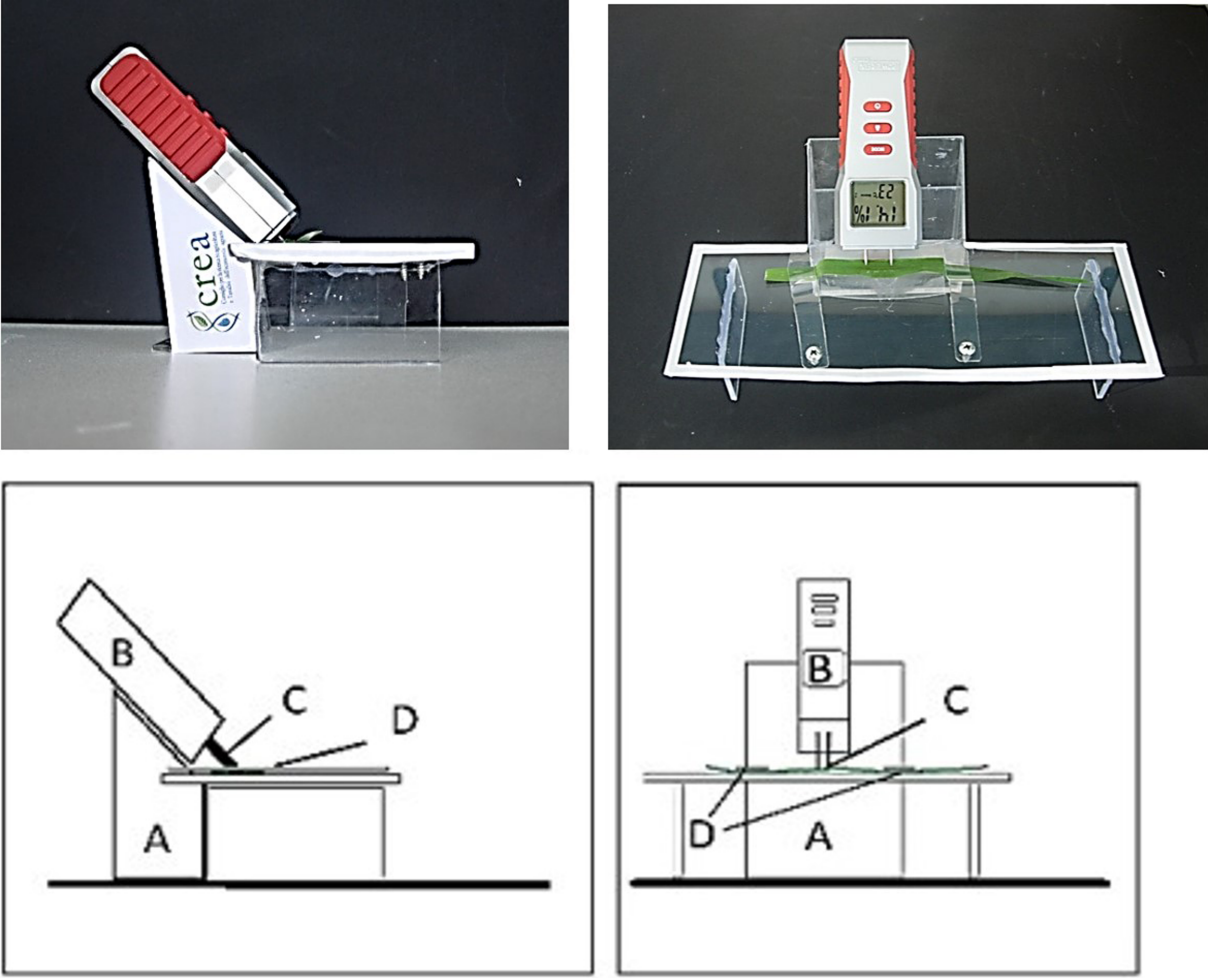
Physical and schematic layout of the leaf moisture meter positioned on the home-made plastic support with a slope of 30° to the leaf housing (**a**). The moisture meter (**b**) has two electrodes that are 1.5 cm apart (**c**) that lightly pierce the leaf, with the leaf held down at the ends using two rotating plastic clamps (**d**)

Moisture meter readings (MMRs) were performed using a wood moisture meter (Profi+; *Powerfix*,) that costs about €10. This has two electrodes that are 1.5 cm apart and is a resistance meter that is designed to measure in direct current. Its use differs from other clamps that completely cross the leaf [26], because these electrodes lightly pierce the leaf. The declared operating voltage is 15 mA (9 V), with resolution of 0.1%. The moisture meter displays the sample temperature and the nominal “percent humidity”. A ‘MODE’ button covered the range from 1 (e.g., for birch, cherry, or other wood samples) to 6 (for brick samples), which can be set according to different materials. Here, Mode 1 had an arbitrary scale from 6 to 44% weight and this was used to analyse both the hydrated fresh and dried re-humidified leaves. The electrodes were applied along the main vein on the adaxial side of the leaves. To apply the same pressure on all of the leaves, the moisture meter was mounted on a specific ‘home-built’ plastic support with a slope of 30° to the leaf housing (Fig. 7). The middle part of the leaf was carefully laid flat and clamped at the ends, using two rotating plastic clamps. Measurements were made at room temperature (27 °C) and 40% relative humidity for: (a) fresh fully-hydrated leaves; (b) dried re-humidified leaves (dried leaves after equilibration for 3 days at 90% relative humidity); and (c) partially dehydrated fresh leaves.

Ten leaves extracted from water or humid air were rapidly blotted dry on absorbent paper and processed for measurement of the MMR. After taking the MMR, the leaves were weighed. The time necessary to analyse each sample was about 1 min. Measurements were repeated every 5 min on two expanded leaves of each genotype, to also analyse the MMR changes with time.

The dry weight (DW) of all of the leaf samples was obtained by drying them in an oven at 105 °C for 12 h. The water content of the leaves was expressed on a DW basis and was calculated according to Eq. (1):

1 $$ {\mathrm{gH}}_2\mathrm{O}/\mathrm{gDW}=\left(\mathrm{FW}-\mathrm{DW}\right)/\mathrm{DW} $$

This was used to normalise the MMR (MMRnorm) to the tissue water content per unit dry matter.

### Moisture meter calibration

To estimate the resistances that corresponded to the values displayed by the moisture meter (at the laboratory of Palazzo Sistemi Elettronici [PSL] S.r.L., Foggia, Italy), the empirical dependence of the MMR on the electrical resistance was examined at 20 °C. A flux millivoltmeter (8842A; Fluke) and a resistivity box (RD-1000; Monacor; with 1% error) were mounted in parallel to the electrodes. Increasing resistances were applied and the corresponding direct current voltage signal and MMRs were recorded.

### Statistics

One-way analysis of variance (ANOVA) was used to test the differences among the means, for the two wheat varieties and for the two leaf expansion conditions. Tukey’s tests and Student’s t-tests were used for comparisons of the means. Regression analysis was performed to define any associations between the variables.

## Data Availability

The datasets used and/or analysed during the current study are available from the corresponding author on reasonable request.

## Abbreviations

ANOVA: One-way analyses of variance
DW: Dry weight
MMR: Moisture meter reading
MMRnorm: Normalised moisture meter reading
